# Sleep and Risk of Multiple Sclerosis: Bridging the Gap Between Inflammation and Neurodegeneration via Glymphatic Failure

**DOI:** 10.3390/brainsci15070766

**Published:** 2025-07-19

**Authors:** Mariateresa Buongiorno, Carmen Tur, Darly Milena Giraldo, Natalia Cullell, Jerzy Krupinski, Roberta Lanzillo, Gonzalo Sánchez-Benavides

**Affiliations:** 1 Neurology Department, Vall d’Hebron University Hospital, 08035 Barcelona, Spain; 2 Vall d’Hebron Research Institute, 08035 Barcelona, Spain; ctur@cem-cat.org; 3 Fundació per a Docència i Recerca, Mútua Terrassa, 08221 Terrassa, Spain; 4 Department of Neurology, Fundació Assistencial Mútua Terrassa, 08221 Terrassa, Spain; 5 Faculty of Science and Engineering, Department of Life Sciences, Manchester Metropolitan University, Manchester M15 6BX, UK; 6 Multiple Sclerosis Clinical Care and Research Centre, Federico II University Hospital of Naples, 80131 Naples, Italy; roberta.lanzillo@unina.it; 7 Department of Neurosciences, Reproductive Sciences and Odontostomatology, Federico II University, 80131 Naples, Italy; 8 Barcelonaβeta Brain Research Center (BBRC), Pasqual Maragall Foundation, 08005 Barcelona, Spain; 9 Hospital del Mar Research Institute, 08003 Barcelona, Spain; 10 Centro de Investigación Biomédica en Red de Fragilidad y Envejecimiento Saludable (CIBERFES), 28029 Madrid, Spain

**Keywords:** multiple sclerosis, glymphatic system, sleep, inflammation, neurodegeneration

## Abstract

Epidemiological studies identified insufficient and poor-quality sleep as independent risk factors for multiple sclerosis (MS). The glymphatic system, active during slow-wave sleep, clears brain waste through perivascular astrocytic aquaporin-4 (AQP4) channels. The presence of antigens induces a transient, physiological lowering of glymphatic flux as a first step of an inflammatory response. A possible hypothesis linking infection with the Epstein–Barr virus, a well identified causal step in MS, and the development of the disease is that mechanisms such as poor sleep or less functional AQP4 polymorphisms may sustain glymphatic flow reduction. Such chronic glymphatic reduction would trigger a vicious circle in which the persistence of antigens and an inflammatory response maintains glymphatic dysfunction. In addition, viral proteins that persist in demyelinated plaques can depolarize AQP4, further restricting waste elimination and sustaining local inflammation. This review examines the epidemiological evidence connecting sleep and MS risk, and the mechanistic findings showing how poor sleep and other glymphatic modulators heighten inflammatory signaling implicated in MS pathogenesis. Deepening knowledge of glymphatic functioning in MS could open new avenues for personalized prevention and therapy.

## 1. Introduction

Multiple sclerosis (MS) is more than a purely immune-mediated demyelinating disease; it sits at the crossroads of viral persistence, sleep biology, inflammation, and brain-waste clearance. In this review, first, we review the epidemiological and molecular evidence that links Epstein–Barr virus (EBV) infection, genetic risk, and sleep loss to the onset of MS. Second, we outline the physiological roles of sleep, especially slow-wave sleep. Third, we examine how the glymphatic pathway operates, what fails in chronic inflammation, and how those failures amplify brain injury, drawing lessons from MS and other disorders, such as Alzheimer’s disease (AD). Finally, we discuss modulators of the system, from non-modifiable aquaporin-4 (AQP4) polymorphisms to modifiable factors such as sleep quality, nocturnal blood-pressure patterns, and pharmacological and non-pharmacological interventions.

## 2. Multiple Sclerosis Risk Factors

Multiple sclerosis (MS) is a chronic, immune-mediated disorder of the central nervous system in which focal demyelination co-exists with diffuse neurodegeneration. Twin and family studies indicate that heritability explains roughly one-quarter of susceptibility, with the class II allele HLA-DRB1*15:01 exerting the largest single genetic effect [1,2]. However, genes alone do not determine who develops the disease. A compelling body of epidemiological work shows that specific environmental exposures are required to convert genetic risk into clinical MS.

The clearest trigger is infection with EBV. The role of EBV in the pathogenesis of MS was proposed four decades ago [3], and recent compelling evidence demonstrated this association. In an elegant prospective analysis of more than ten million U.S. service members, virtually every individual who later developed MS seroconverted to EBV before symptom onset, and seropositivity multiplied risk more than thirty-fold [4]. How EBV interfaces with host immunity is still debated, but most frameworks invoke a chronic, low-grade inflammatory state that lowers the threshold for CNS-directed autoimmunity. Recent spatial imaging work has strengthened this link. High-dimensional microscopy of progressive MS autopsy tissue shows EBV markers (EBNA1, LMP1) concentrated inside lesions, where EBV-positive cells sit near reactive astrocytes, microglia, and neurons, and co-localize with zones of disrupted blood-brain barrier endothelium. These observations place the virus directly within the lesion microenvironment and suggest that EBV persistence may sustain local glial–neuronal stress and vascular leaks, providing a structural bridge between the epidemiologic trigger and the chronic, compartmentalized inflammation characteristic of MS [5].

Several modifiable factors consistently modulate risk. Prospective cohorts link low serum 25-hydroxy vitamin D to higher MS incidence [6], while cigarette smoking increases both the likelihood of disease and the pace of neuronal loss [7]. Excess body-mass index during adolescence also elevates risk, possibly through adipokine-driven inflammation [8]. Finally, night-shift or rotating schedules undertaken early in life carry roughly a two-fold hazard, an effect attributed to circadian disruption [9,10]. Sleep itself has recently emerged as an independent determinant. In a Swedish population-based case-control study of 2075 cases and 3164 controls, sleeping fewer than seven hours per night between the ages of 16 and 19 was associated with a 40% rise in MS odds, and low subjective sleep quality raised risk by about 50% [11]. A second analysis using 1253 cases and 1766 controls demonstrated that inadequate sleep short duration and poor quality interacted additively with HLA-DRB1*15:01, tripling to sextupling risk relative to well-rested non-carriers [12]. These findings lend biological plausibility to the idea that sleep loss, which is known to amplify immune activation and compromise the antiviral response, have an impact in MS etiology. Experimental sleep restriction in humans activates nuclear factor- κB (activator protein 1 and signal transducer and activator of transcription family proteins), boosts circulating IL-6 and TNF-α, and shifts T-cell cytokines toward a more inflammatory profile, thereby heightening immune activation. The same body of work shows that shortened sleep suppresses type-I interferon signaling, weakens antibody responses to influenza and hepatitis vaccines, and almost doubles the risk of catching common cold viruses, underscoring how sleep loss affects antiviral defense [13].

## 3. Sleep Physiological Functions in the Brain

Sleep is a basic need shared by nearly every mammal. During non-REM sleep the cortex produces slow waves that permit synapse restoration, saving energy and preventing circuits from saturating [14]. At the same time, brief bursts of activity replay the events experienced while awake, consolidating new memories into long-term stores [15]. In slow-wave sleep, the hippocampus replays recent events while slow waves, spindles, and ripples line up to move this replay to the cortex and strengthen the right links. At the same time, overall connection strength is gently reduced so circuits do not overload. With each cycle, memories shift toward long-term cortical stores, dropping fine details but keeping the main information. This explains why solid sleep supports learning and keeps brain networks balanced [16]. Behind these network events, a marked molecular switch occurs, and thousands of genes change their output between wake and sleep. Genes that build membranes, traffic vesicles, and repair DNA rise when the brain is asleep, while transcripts that drive stress responses and high energy use dominate the waking state [17,18]. If sleep is shortened, the switch flips the wrong way: circadian rhythms fade, inflammatory genes surge, and circulating cytokines increase [13,19].

Mounting evidence indicates that the efficiency of the brain’s nocturnal clearance pathway links to key graphoelements of non-REM sleep. Reduced clearance is associated with lower slow-wave activity (SWA), diminished spindle density, and weaker phase coupling changes that predict poorer memory consolidation overnight [20]. The molecular waste removed by this pathway includes phosphorylated tau and amyloid-β, and early cortical tau deposition has already been linked to selective losses of fast spindles and hippocampal-cortical communication before amyloid positivity emerges [21]. Imaging markers of clearance efficiency decline stepwise from healthy controls to individuals with isolated REM sleep behavior disorder and to patients with Parkinson’s disease, suggesting that impaired waste removal extends its influence beyond AD to other neurodegenerative conditions [22]. During the deepest stages of non-REM sleep the space between brain cells widens, cerebrospinal fluid flows through perivascular pathways tunnels, and metabolic waste is cleared far faster than in the waking brain [23]. This sleep-activated glymphatic flow, and how its failure might connect poor sleep to persistent inflammation and neurodegeneration in MS, is the focus of the next sections.

## 4. Glymphatic System

The glymphatic system is a brain-wide pathway that moves cerebrospinal fluid (CSF) from peri-arterial spaces into the parenchyma, mixes it with interstitial fluid, and directs the mixture toward peri-venous routes for clearance. Astrocytic end-feet that surround cerebral vessels are rich in the water channel AQP4. This polarized (asymmetric, well-organized localization of channels) AQP4 arrangement is essential, because it lets CSF flow through the perivascular sheath and into the interstitium [24,25].

Flow is not constant. Arterial pulsations, respiration, and slow vasomotor waves act as pumps, but the most powerful switch is behavioral state. During deep non-REM sleep, cortical slow waves reduce cell volume and widen the extracellular space by about 60%, lowering resistance and doubling bulk fluid movement compared with quiet wakefulness [23]. In rodents, blocking slow waves or keeping animals awake for a single night cuts glymphatic flux by half; in humans, diffusion-weighted MRI and intrathecal tracer studies show the same sleep dependence [26,27].

Several factors modulate the system performance. Age and vascular stiffening dampen arterial driving forces, reducing clearance [28]. Genetic or acquired loss of AQP4 polarity, as seen after traumatic brain injury, in the presence of some AQP4 single-nucleotide variants, or when astrocytes become reactive, slows solute movement and leaves macromolecules behind [29]. Head position, systemic blood pressure, and even breathing depth can fine-tune the rate, while chronic inflammation narrows perivascular spaces through cellular swelling and pericyte activation [30].

These factors have direct relevance for MS. Experimental autoimmune encephalomyelitis mice models show early AQP4 depolarization and a 40–50% fall in glymphatic inflow [31], and patients with MS show reduced glymphatic function—as measured by diffusion along perivascular spaces (DTI-ALPS) [32,33,34,35]—and other proxies of glymphatic dysfunction such as enlarged perivascular spaces [36]. Carotenuto et al. examined 71 individuals with MS (49 relapsing–remitting, 22 progressive) and 32 matched controls. Glymphatic functioning, as measured by DTI-ALPS, was lower in the MS group overall, fell further in progressive subtype, and showed a clear clinical gradient. Lower ALPS values correlated with higher disability scores (r = 0.45), longer disease duration, greater white- and grey-matter lesion burden, deeper cortical and subcortical atrophy, and microstructural injury in normal-appearing white matter. Notably, the decline in ALPS was steepest during the first four years after onset and then plateaued, suggesting that impaired clearance is an early, self-propagating process rather than a late by-product of tissue loss [33]. The next section examines how sustained inflammation in MS might both result from, and further worsen, this glymphatic slowdown.

## 5. Chronic Inflammation and Glymphatic Dysfunction in MS

### 5.1. The Enduring Inflammation Model

Inflammation in MS does not simply damage myelin; it progressively reshapes the brain’s fluid-clearance network. The “enduring inflammation” model derived from rodent and human data proposes a series of events initiated by a new antigen. Within this model, Mogensen and colleagues [37] outline a three-step response of brain fluid dynamics to an acute inflammatory hit. **Phase I** begins within minutes: the choroid plexus briefly overproduces CSF, creating a “flush” that can carry newly released cytokines and antigens from activated glia toward peripheral lymph nodes. Astrocytes up-regulate AQP4 at the same time, but these extra channels are scattered across the cell surface rather than confined to the vascular end-feet, so the surge of fluid does not translate into efficient glymphatic flow. In **Phase II** the system stalls. Waste products, signaling molecules, and slow drainage cause perivascular and meningeal swelling, leading to tissue oedema. Finally, during **Phase III** the oedema descends and CSF production returns to baseline. However, astrocytic and microglial changes may persist, leaving the glymphatic pathway chronically less effective even after acute inflammation has resolved.

In most infections this process resolves within hours, but when the antigen persists, as can occur with latent EBV in meningeal B cells, reactive astrocytes lose their end-feet polarization of AQP4 water channels, arterial pulsations weaken, and leukocytes accumulate in the same perivascular tunnels that normally drive CSF into the parenchyma. Experimental work had demonstrated that EBV proteins can depolarize AQP4, compounding the mechanical blockade [38]. Together these imaging and molecular findings reinforce the feed-forward loop proposed above: early antigen persistence slows glymphatic flow; the resulting stagnation fuels inflammation and structural damage, which in turn further impairs clearance. With time, the transient bottleneck consolidates into an enduring low-flow state. Perivascular cuffs fibrose, mislocalized AQP4 becomes permanent, and waste products accumulate. Post-mortem studies reveal plumes of demyelination spreading out from central veins [39,40], exactly the route glymphatic fluid would normally take to exit the brain. Reduced glymphatic drainage permits pro-inflammatory cytokines and other neurotoxic factors to persist in perivenous spaces, maintaining microglial activation and perpetuating the inflammatory process [41].

A recent proteomic investigation in amnestic mild cognitive impairment (MCI) patients assessed glymphatic performance with serial 3T gadolinium-enhanced MRI, and related regional clearance half-times to 7000 cerebrospinal-fluid proteins quantified on an aptamer panel. Top associations were verified in an independent group of MCI cases and controls. Only seven proteins displayed consistent, region-wide correlations with clearance speed, and pathway enrichment highlighted B-cell activation, JAK/STAT, histamine and angiotensin signaling—canonical inflammatory routes. Faster wash-out coincided with lower concentrations of these immune markers, whereas slower clearance aligned with an inflammatory profile, supporting an inverse relationship between immune activation and glymphatic efficiency [42]. Although conducted in prodromal AD, these findings reinforce the premise that sustained inflammation hampers brain waste transport and, in turn, is perpetuated by the resulting flow stagnation. This interaction is central to the “enduring inflammation” loop that we propose is also relevant for MS.

Sleep loss intensifies every step in this cascade. Deep non-REM oscillations are the principal pump for glymphatic inflow and reducing sleep by just two hours a night halves flow in animals and boosts inflammatory transcripts in humans [13,23]. This evidence aligns with the epidemiological evidence on the increased risk of MS in adolescents who report sleeping fewer than seven hours or poor-quality sleep [11,12]. In MS patients, sleep disorders are common (up to 75% [43]). Restless-legs syndrome affects roughly 15–58% of patients and obstructive sleep apnoea 7–58% [44]. Notably, these disturbances have been associated with reduced slow-wave power and larger lesion volume, implying that disease activity and sleep disruption reinforce one another [44,45]. Evidence suggests that sleep problems in MS are both persistent across the course of disease and capable of flaring around relapses. In a two-year cohort of relapsing MS patients, more than half of the participants scored above the Pittsburgh Sleep Quality Index (PSQI) insomnia threshold at every visit; the proportion with poor sleep quality and the mean PSQI score showed no significant change over 24 months, indicating a stable, chronic disturbance [46]. Another longitudinal study that followed newly diagnosed relapsing-remitting MS cases for two years likewise found that insomnia category (no, sub-threshold, or clinical) remained unchanged; sleep complaints persisted even though none of the participants experienced a relapse during that interval [47]. Regarding relapses, case-control data collected at the time of an acute exacerbation showed poor sleep quality in 87.5% of relapse cases versus 50% of matched remission controls (OR 1.75), suggesting that disturbed sleep can exacerbate during, and may even precipitate, clinical relapses [48].

### 5.2. Neuromyelitis Optica as a Prototype of Enduring Inflammation via Water-Channel Loss

Neuromyelitis optica (NO) illustrates what happens when AQP4 becomes the direct target of immunity. Serum IgG1 antibodies against AQP4 bind the channel’s extracellular loops, activate complement, and trigger antibody-dependent cytotoxicity [49]. Within minutes, astrocytes in the spinal cord and optic nerve detach their end-feet, lose polarized AQP4, and swell, while the choroid plexus briefly boosts CSF output (Phase I according to the Mogensen et al. enduring inflammation model) [37]. If pathogenic antibody persists, the system shifts to Phase II. Complement pores and neutrophil proteases perforate astrocyte membranes, perivascular cuffs fill with protein-rich oedema, and meningeal lymphatic drainage slows markedly. Tissue water rises, yet directional glymphatic flow collapses because any newly made AQP4 is scattered across the astrocytic surface instead of clustering at end-feet; complement activation initiated by AQP4 autoantibodies perpetuates astrocytic injury and sustains a localized pro-inflammatory milieu [50]. Resolution (Phase III) is only partial. Even after oedema subsides, many astrocytes lack their perivascular AQP4 cap, basement membranes scar and narrow the tunnels, and tracer studies show chronically blunted glymphatic inflow and efflux. NO thus provides a “pure” example of enduring inflammation driven by primary AQP4 failure: antibodies disable the channel, oedema follows, and long-term glymphatic dysfunction both reflects and reinforces astrocyte injury. The sequence highlights how tightly water transport, immune surveillance, and waste clearance are coupled in the central nervous system.

## 6. Glymphatic Failure as the Bridge Between Inflammation and Neurodegeneration

In our view, the glymphatic system plays a key role in the observed association between poor sleep, inflammation, and neurodegeneration, not only in MS but also in other diseases such as AD.

### 6.1. Neurodegeneration in MS

Neurodegeneration and progressive tissue loss in MS seems to relate to local inflammation inside the brain and the re-programming of support cells. After the acute relapse declines, disease-associated microglia and reactive astrocytes up-regulate and secrete complement components, iron-handling proteins such as ferritin heavy chain, oxidative-stress-related molecules, and pro-inflammatory cytokines that can perpetuate local neurodegeneration [51]. These signals affect axonal mitochondria, let sodium and calcium flood in, and carve out the slow-growing “iron-rim” lesions that predict later disability. At the same time, oligodendrocyte precursor cells switch to an inflammatory mode; they stop maturing and fail to wrap damaged axons, leaving nerve fibers in distress [52]. Because glymphatic flow is already reduced by chronic astrocyte swelling and misplaced AQP4 channels, the toxic by-products of this immune activity remain in the tissue, tightening the spiral from inflammation to neurodegeneration.

### 6.2. Neurodegeneration in AD

AD is the most common form of dementing diseases. It is a progressive disorder where neurons and synapses gradually degenerate, leading to cognitive decline and memory loss. This process is driven by an abnormal buildup of Aβ plaques and tau protein tangles in the brain. These aggregates disrupt synaptic communication, cause neuronal dysfunction, and are tightly linked with a chronic inflammatory response. Microglial cells, the brain’s immune sentinels, react by releasing pro-inflammatory cytokines, complement proteins, and reactive oxygen species, which further damage surrounding tissue [53,54]. Recent studies have highlighted how the immune system plays a role beyond the brain. T lymphocytes infiltrate the brain and secrete cytokines such as interferon-gamma and interleukin-17, worsening the damage and promoting tau pathology [55,56]). Vascular dysfunction, including blood-brain barrier leakage and cerebral amyloid angiopathy, worsens this cycle by impairing waste clearance and allowing more inflammatory mediators to enter the brain [57]. The result is a self-reinforcing loop of toxic protein accumulation, immune activation, and neuronal injury, creating a “toxic milieu” that undermines synaptic integrity and leads to atrophy of key brain regions like the hippocampus and cortex [58]. This chain of events shares striking similarities with the low-grade inflammation and impaired glymphatic flow seen in MS, suggesting that sleep disturbances and glymphatic dysfunction could be common pathways linking inflammation and neurodegeneration in both diseases.

### 6.3. Bridging the Gap: Glymphatic Modulators 

There is a clear link between a chronic inflammatory response and neurodegeneration. Alghanimy and colleagues recently proposed that MS could be seen as a “glymphaticopathy” [41]. We agree with this view and propose, accordingly, that known glymphatic modulators (both non-modifiable and modifiable) may have an impact on the process, shaping the pathogenesis and progression of MS and other neurodegenerative diseases. The global model would be mostly universal, i.e., an antigen enters the system, provokes inflammation and hampers glymphatic clearance, which in turn worsens the downstream effects of inflammation, provoking a cascade that finally leads to neurodegeneration. The individual genetic susceptibility, the moment in life in that infection occurs, glymphatic modulators, and other unknown factors would determine the specific disease and progression. As EBV infection in young individuals may lead to MS in the presence of deleterious variants of HLA-DRB1*15:01 and poor sleep [12], herpes simplex virus-1 (HSV1) and other infections and antigens seems related to AD pathological events. Prior infection with HSV-1, influenza, or varicella zoster virus raises later odds of AD and related dementias [59]. More strikingly, a recently published “natural experiment” found that the recombinant shingles vaccine reduced new dementia diagnoses by 20% over eight years [60]. Meta-analyses report similar protection from influenza and pneumococcal vaccines [61]. Airborne pollutants add non-infectious evidence to the model. Long-term exposure to fine particulate matter (PM2.5) accelerates microglial activation and increases incident dementia by roughly 25% in population cohorts [62], a finding confirmed in a meta-analysis of six million participants [63]. Similarly, fungi co-localize with plaques and tangles and keep innate immunity activated [64]. Whether the downstream disease is MS, AD, or another disorder may depend on genetics, age at exposure, and many other factors, including glymphatic modulators. Indeed, recent evidence suggests that MS patients may be at higher risk of developing AD pathology, being an overlapping between both diseases [65]. Novarella et al. found that plasma neurofilament light chain (a marker of neurodegeneration) is associated with biomarkers of amyloid accumulation in elderly people with MS, which are associated with cognitive impairment [66].

Among glymphatic modulators, AQP4 single nucleotide polymorphisms (SNPs) would represent the main non-modifiable factor. In cognitively healthy adults, carriers of the minor allele at *rs72878776* show increased cortical amyloid-β deposition associated with poorer sleep [67]. Other SNPs, *rs9951307* and *rs3875089*, are associated with a faster increase in white-matter free water, a diffusion metric that tracks impaired interstitial clearance, and with memory decline over five years, whereas the protective *rs72878794* allele shows the opposite pattern [68].

The main potentially modifiable glymphatic factors are sleep micro- and macro-structure, vascular pulsatility, and pharmacological modulation. During the slow-wave (<4 Hz) sleep phase, cortical neurons fire in large, synchronous patterns that are coupled to low-frequency changes in arteriole diameter [69]. In mice, each slow wave is followed within seconds by a CSF pulse and a measurable increase in solute clearance; when slow-wave activity is fragmented, the flow response quickly disappears [70]. In addition, when nocturnal blood pressure fails to dip, or even rises, glymphatic clearances seem to diminish. Recent evidence using DTI-ALPS shows that non-dipping patterns relates to worse glymphatic functioning [71]. Mechanistic data in mice indicate that arterial wall pulsations are the principal motor of cerebrospinal-fluid ingress; hypertension amplifies retrograde waves, cutting net flow by almost half [72]. These observations suggest that “nondipping” and “riser” night patterns compromise the nightly vascular pulse that drives clearance.

Pharmacology offers two complementary ways to restore sleep haemodynamic profile. First, β-adrenergic antagonists that readily cross the blood–brain barrier, such as propranolol and carvedilol, shrink astrocytic volume, expand the interstitial space, and are associated with higher CSF Aβ42 in non-demented adults [73]. Second, dual orexin receptor antagonists deepen and consolidate non-REM sleep, indirectly boosting glymphatic inflow. Daridorexant 50 mg reduces wake-to-wake transitions, lowers cortical β-power, and increases delta activity after three months of treatment [74], while lemborexant 5–10 mg shows similar improvements in total sleep time and maintenance in two phase-3 trials [75]. Beyond pharmacology, 40 Hz sensory stimulation has emerged as a non-invasive means to reinforce glymphatic pumping. In aged APP/PS1 mice, one hour of closed-loop light–sound stimulation at 40 Hz each night deepened slow-wave activity, repolarized perivascular AQP4, increased peri-arterial CSF influx by roughly 40%, and halved cortical amyloid within seven days [76]. A complementary human study delivered phase-locked 40 Hz auditory bursts during stage N2 sleep; the stimulation preserved sleep architecture, entrained frontal gamma oscillations, and produced a measurable rise in ventricular CSF pulsatility on fMRI without next-day cognitive cost [77]. Together, these findings suggest that targeted gamma entrainment can reactivate the vascular-astroglial pump when endogenous slow waves or arterial pulsations are blunted, offering a practical adjunct for restoring glymphatic.

## 7. Discussion and Future Directions

**Figure 1:**
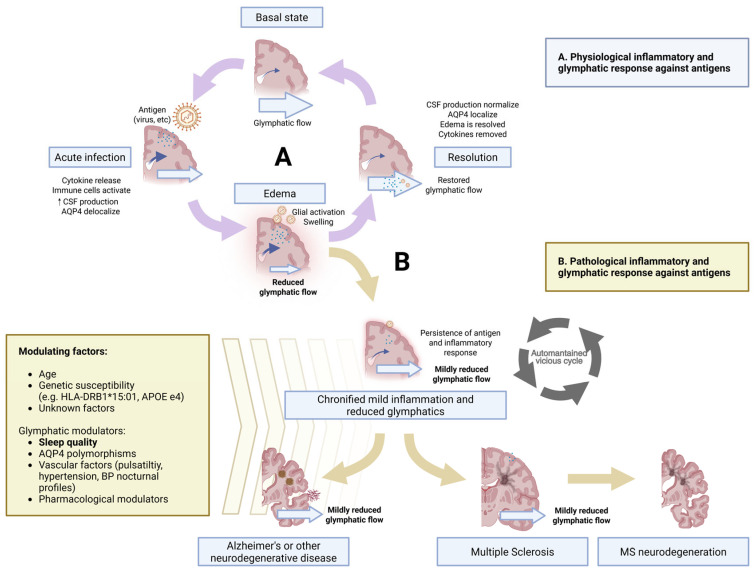
Inflammatory response and glymphatic modulators in the etiology and progression of MS and other diseases. https://BioRender.com/gu90j7t (accessed on 16 June 2025).

The evidence presented converges on the following sequence: a latent or recurrent antigen (EBV in MS) initiates a compartmentalized inflammatory response. That response depolarizes AQP4 and narrows perivascular spaces, slowing glymphatic flow; poor or fragmented slow-wave sleep prevents nightly recovery of clearance and retained antigens and cytokines then fuel further inflammation, locking the system into an “enduring-inflammation” loop that ultimately damages axons and neurons. The same framework helps explain why chronic sleep loss, vascular stiffening, and airborne pollutants and other antigens promote other neurodegenerative diseases, such as AD and related dementias (see Figure 1).

This model points to four therapeutic points that may help to prevent or ameliorate MS and other neurodegenerative disorders in which chronic inflammation alters glymphatic function:

1. Remove or silence the antigen. EBV vaccination, antiviral strategies, and broader pathogen-based prevention (e.g., shingles, influenza, pneumococcal vaccines) merit prospective trials that include imaging markers of glymphatic function.
2. Ensure and restore deep sleep architecture. Behavioral sleep optimization, treatment of sleep-disordered breathing, and if needed, pharmacological consolidation with dual orexin-receptor antagonists such as lemborexant or daridorexant may be evaluated for their ability to boost slow-wave power and, in turn, glymphatic inflow. Strategies globally improving sleep quality may also positively impact the entire physiological process that supports memory consolidation.
3. Normalize nocturnal haemodynamics. Although not so relevant in MS due to age of onset, controlling riser or non-dipping sleep blood-pressure patterns, particularly with blood–brain barrier-permeable β-blockers that also shrink astrocytic volume, could enhance perivascular pulsatility and widen interstitial space.
4. Repolarize or augment AQP4 function. Small molecules that stabilize AQP4 at end-feet may help polarization and glymphatic flow.
5. Non-pharmacological frequency-based interventions. Neuromodulation therapies enhancing glymphatic functioning, such as 40 Hz multisensory stimulation and others, may durably boost clearance and dampen inflammation.

Future studies combining overnight sleep EEG, blood-pressure monitoring, and advanced diffusion MRI would test how these interventions influence clearance and clinical outcomes. As glymphatic impairment emerges early, often preceding overt neurodegeneration, multimodal, time-sensitive strategies may help slow disease progression and address its underlying mechanisms.

## Data Availability

No new data was generated for this paper.
